# Effectiveness and Adverse Events of Cyclophosphamide, Vincristine, and Prednisolone Chemotherapy in Feline Mediastinal Lymphoma Naturally Infected with Feline Leukemia Virus

**DOI:** 10.3390/ani12070900

**Published:** 2022-03-31

**Authors:** Supita Sunpongsri, Attawit Kovitvadhi, Jatuporn Rattanasrisomporn, Viphavee Trisaksri, Nichakorn Jensirisak, Tassanee Jaroensong

**Affiliations:** 1 Kasetsart University Veterinary Teaching Hospital, Faculty of Veterinary Medicine, Kasetsart University, 50 Ngamwongwan Rd., Lat Yao, Chatuchak, Bangkok 10900, Thailand; supita.su@ku.th (S.S.); fvetvvt@ku.ac.th (V.T.); nichakorn.je@ku.th (N.J.); 2 Department of Companion Animal Clinical Sciences, Faculty of Veterinary Medicine, Kasetsart University, 50 Ngamwongwan Rd., Lat Yao, Chatuchak, Bangkok 10900, Thailand; fvetjpn@ku.ac.th; 3 Department of Physiology, Faculty of Veterinary Medicine, Kasetsart University, 50 Ngamwongwan Rd., Lat Yao, Chatuchak, Bangkok 10900, Thailand; fvetawk@ku.ac.th

**Keywords:** adverse events, cat, COP chemotherapy, feline leukemia virus, lymphoma

## Abstract

**Simple Summary:**

Feline leukemia virus (FeLV) infection is considered a poor prognostic factor for feline lymphoma. Cyclophosphamide, vincristine and prednisolone (COP) chemotherapy is a standard protocol treatment of feline lymphoma. This retrospective study involved 92 cats diagnosed with mediastinal or mediastinal plus other anatomical sites of lymphoma and treated with COP chemotherapy. Clinical signs and adverse events were observed after the 1st, 2nd, and 3rd inductions. Clinical signs improved after the 3rd induction of COP chemotherapy. The response rate was 96.74%. The overall median survival time was 338 days (range 62–1057 days). This study found that cats aged <4 years had longer survival than those aged at least 4 years. Anemia (before COP), azotemia (after 2nd induction) and elevated alanine aminotransferase (after 1st induction) was associated with an increased chance of mortality.

**Abstract:**

Feline leukemia virus (FeLV) infection is considered a poor prognostic factor for feline lymphoma. This study investigated the prevalence of cats suffering from feline lymphoma with natural infection of the feline leukemia virus, as well as clinical signs, adverse events, and survival time after cyclophosphamide, vincristine and prednisolone (COP) chemotherapy. This retrospective study involved 92 cats diagnosed with mediastinal or mediastinal plus other anatomical sites of lymphoma and treated with COP chemotherapy. FeLV-antigen-positive was observed in all cats. Clinical signs and adverse events were observed after the 1st, 2nd, and 3rd inductions. Clinical signs improved after the 3rd induction of COP chemotherapy. The response rate was 96.74% (81.52% complete response, 15.22% partial response, and 3.26% no response). The overall median survival time was 338 days (range 62–1057 days). The overall response rate and median survival time of cats with feline lymphoma that were FeLV-antigen-positive and treated with COP chemotherapy were higher than from other studies. This study found that cats aged <4 years survived longer than those aged at least 4 years. Anemia (before COP), azotemia (after 2nd induction), and elevated alanine aminotransferase (after 1st induction) were associated with an increased chance of mortality.

## 1. Introduction

Lymphoma is one of the most common tumors leading to deaths in both humans and companion animals worldwide [1,2]. Lymphoma represents 83% of canine [3] and 90% of feline [4] hematopoietic-origin tumors. Various associations have been reported between lymphoma, age, sex, neutering status, and breed [5,6,7,8,9,10,11]. Lymphoma can be classified by anatomical types into multicentric, mediastinal, renal, hepatic, nodal, and alimentary [12], which can be characterized as solitary, diffuse, or multifocal [13].

Progressive FeLV-infected cats have around a 60-fold greater risk of developing lymphoma compared with non-infected cats. Mediastinal lymphoma (especially in the pre-vaccination era in high prevalence areas) is the most common form of FeLV-associated lymphoma, followed by multicentric lymphoma [11,14,15].

Clinical signs from asymptomatic to severe vary for cats with retroviral infection and lymphoma. Severe clinical signs can result in acute death [16]. Respiratory distress from pleural effusion is usually associated with feline mediastinal lymphoma [17].

There are many chemotherapy protocols available for the treatment of feline lymphoma [4,7,8,18,19,20,21]. Complete and partial response rates as well as median survival rate were reported to be not significantly different between the two protocols: COP (cyclophosphamide, vincristine and prednisolone) and Madison–Wisconsin [7].

The adverse events of chemotherapy usually occurred within 24–48 h or with acute delay within 2–14 days after treatment [19,22]. To our knowledge, the association between adverse events and survival time has not been reported.

Investigation of the onset and duration of clinical signs and adverse events from COP chemotherapy may benefit feline lymphoma treatments and prognosis. This study aimed to identify the prevalence of cats naturally infected with FeLV in feline lymphoma and to investigate the clinical signs, adverse events, and survival time of COP chemotherapy in feline lymphoma.

## 2. Materials and Methods

### 2.1. Clinical Evaluation and Treatment

This research involved a retrospective study that considered the medical records of client-owned cats diagnosed with mediastinal lymphoma at the Kasetsart University Veterinary Teaching Hospital (Bangkok, Thailand). The database was compiled by reviewing the cases of hospital visits between June 2019 and June 2020.

Various data on the treated cats were recorded: age, sex, breed, reproductive status, clinical signs (Supplementary Table S1; modified according to Collado [16]), retroviral status, adverse events of chemotherapy (Supplementary Table S2; modified according to the Veterinary Cooperative Oncology Group-Common Terminology Criteria for Adverse Events (VCOG-CTCAE v2) [23]), response to chemotherapy, remission, and survival time. The clinical signs were observed and evaluated in cats to obtain a clinical score (CS) from the day of diagnosis to after 3rd induction. CSs were classified into three clinical groups (CG): CG1, with no clinical signs (asymptomatic); CG2, with CS 1–5 (mild disease); and CG3, with CS ≥ 6 (severe disease). The anatomical location of each lymphoma was divided into mediastinal and mediastinal plus other sites.

Inclusion criteria were a cytological or histopathological diagnosis of lymphoma from pleural effusion or cranial mediastinal mass and the presence of a cranial mediastinal mass from thoracic radiography or ultrasonography. FeLV testing was performed using rapid immune-migration (RIM)-based methods (WITNESS^®^). Cats were negative using the test kit FeLV antigen had a whole blood nested polymerase chain reaction (PCR) analysis for FeLV provirus [24]. All cats were treated using the COP protocol according to Teske [8].

During COP chemotherapy, if leukopenia (<3000/μL) or neutropenia (<1500/μL) occurred, the COP chemotherapy was delayed. In cats with leukopenia or neutropenia LEUKOPLUS^®^ (a white blood cell growth factor analogue) a dose of 3–10 μg/kg was administered subcutaneously for three consecutive days. If the adverse events progressed to grade IV, the chemotherapy was terminated and changed to palliative treatment. Cats were excluded if they had been treated with a chemotherapy protocol other than the COP protocol as the first line or if medical records were incomplete.

Between June 2019 and June 2020, 122 cats that had presented for lymphoma and been treated with COP chemotherapy were reviewed. In total, 92 cats met the mediastinal lymphoma inclusion criteria. The median age was 2 years (range 8 months to 11 years). There were 41 neutered males (44.57%), 19 intact males (20.65%), 15 spayed females (16.30%), 11 intact females (11.96%), 2 unknown males, and 4 unknown females. The breeds represented were: 88 domestic shorthairs (95.65%), 2 Persians, and 2 Scottish folds.

### 2.2. Statistical Analysis

Factors analyzed for response to COP chemotherapy were breed, age, sex, retroviral status, clinical signs, and adverse events. The Kaplan–Meier, log rank test and the Cox proportional hazard method were used to estimate survival analysis for which the survival time was calculated from the date of diagnosis to the date of death. Cats were censored if they were alive at the end of the study or if lost to follow-up. A *p*-value < 0.05 was considered statistically significant. All statistical analyses were performed using the R software (R Core Team, 2020) in the RStudio Desktop 1.3.1093 environment with the Rcmdr, Survival and Survminer packages.

## 3. Results

All 92 cats were FeLV-antigen-positive. Tumor anatomical location was mainly recorded only as mediastinal for 78 cats (84.78%) and as mediastinal plus other sites for 14 cats (15.22%).

The number of cats presenting with dyspnea and requiring thoracocentesis before COP chemotherapy was 65 (70.65%). After the induction of first COP chemotherapy, there were 23 cats (25%) that had one or more thoracocentesis performed, while 69 cats (75%) no longer required thoracocentesis. The descriptive data of cats recruited in the study are summarized in Table 1.

As shown in Table 2, the most common clinical signs (Supplementary Table S1) on the day of diagnosis were: respiratory disorders (71.74%), loss of appetite (38.05%), lymphadenomegaly (18.86%), asthenia (25%), dehydration (21.74%), weight loss (14.13%), oral lesion (11.54%), pale mucous membranes (4.35%), neurologic disorders (3.26%), and conjunctivitis (1.09%).

After the 1st induction, common clinical signs were loss of appetite and weight loss. All clinical signs, including conjunctivitis, skin lesions and diarrhea, diminished following the 3rd induction of COP chemotherapy.

According to the current VCOG-CTCAE grading system [23], the adverse effects of COP chemotherapy (Supplementary Table S2) are hematologic toxicity (anemia, leukopenia, neutropenia, azotemia and elevated alanine aminotransferase (ALT)) and signs of anorexia, vomit, or diarrhea, as indicated in Table 3.

Anemia is the most common COP chemotherapy adverse event, followed by leukopenia, azotemia, elevated ALT, and neutropenia. The common adverse events signs were anorexia, vomit, and diarrhea, respectively. The majority of adverse events were grade I except for one cat who had anemia grade IV and four cats who had elevated ALT grade IV after the first induction.

**Figure 1:**
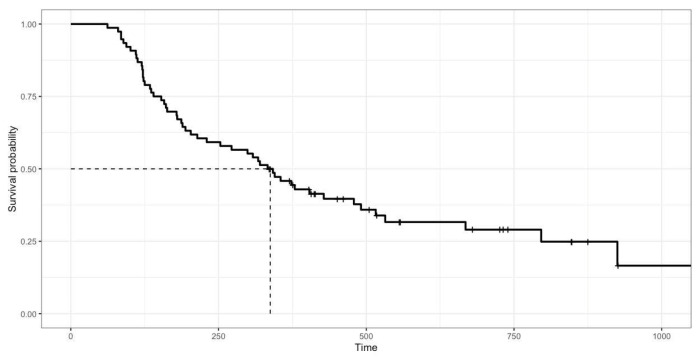
Kaplan–Meier survival curve showing overall median survival of 76 cats with mediastinal lymphoma treated with COP chemotherapy. Median survival time was 338 days (range 62–1057 days).

**Figure 2:**
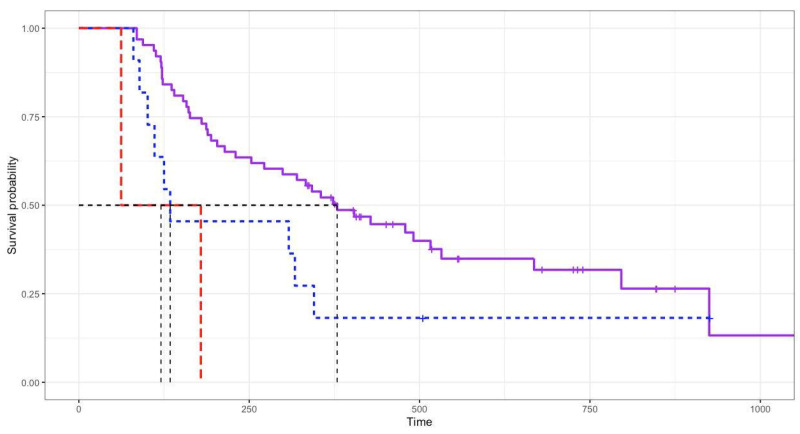
Log rank test analysis showing response to COP chemotherapy. Cats with complete response (MST 379 days; purple line) had significantly (*p* = 0.008) longer survival times than those with partial response (MST 134 days; blue line) and no response (MST 121 days; red line).

Based on the survival analysis of 76 cats, the overall response rate was 96.74% (81.52% complete response, 15.22% partial response, and 3.26% no response). At the end of the study, 52 cats (68.42%) had died and 24 cats (31.58%) were alive (median: 518 days, range 336–1057 days). The overall median survival time (MST) of the 76 cats was 338 days (range 62–1057 days; Figure 1). Cats with a complete response (MST 379 days) had significantly (*p* = 0.008) longer survival times than those with a partial response (MST 134 days) and no response (MST 121 days), as shown in Figure 2. In total, 81 cats had a mean disease-free interval of 231 days (range 12–1017 days).

The median survival times based on sex, reproductive status, retroviral status, anatomical location, and blood transfusion after COP were not different, according to the log rank test analysis of the 76 cats. Cats aged <4 years (MST 373 days) had longer survival times than those aged at least 4 years (MST 212 days) (*p* = 0.01). After the first induction of COP, cats that required thoracocentesis (MST 147 days) had shorter survival times (*p* = 0.002) than those not requiring thoracocentesis (MST 373 days).

Table 4 summarizes the results of the Cox-regression analysis of the 76 cats. Cats with anemia (before COP), azotemia (after 2nd induction) and elevated ALT (after 1st induction) had an increased hazard of death. Through multivariable analysis, cats with anemia before COP (*p* = 0.013; hazard ratio (HR) = 2.5), with azotemia after 2nd induction (*p* = 0.044; HR = 2.3) and with elevated ALT after 1st induction (*p* = 0.014; HR = 2.8) all had a higher risk of death.

## 4. Discussion

Other studies have shown that mediastinal lymphoma occurs in young-aged cats. The median age for cats in the current study was 2 years, which was similar to other studies [7,18,25]. The bimodal age distribution in previous studies had peaks at 1 year and >8 years [7,8,9,26,27]; however, due to the high incidence of FeLV-antigen-positive cats in our research, this did not occur. Notably, domestic shorthair cats were overrepresented (more than 90%) in this study. The high prevalence of this breed might be attributable to the country (Thailand) with high FeLV prevalence and an outdoor lifestyle environment; however, no specific data were collected in this study and so it was not possible to conclude breed predisposition to domestic shorthairs. Male cats had a greater ratio than female cats in this study, with the ratio of 2.1:1 being similar to other studies [5,7,9,10].

However, FeLV-antigen-positive cats presented with clinical signs at the day of diagnosis that related to retroviral infection [16]. The most common were respiratory disorders, loss of appetite, and anorexia, all of which impaired quality of life. All clinical signs except neurological signs decreased after the third induction of COP chemotherapy. This might have been due to the complex pathogenesis and outcomes of retroviral infection or adverse events of COP chemotherapy [22,23].

The current study found that COP chemotherapy, the most popular protocol for feline lymphoma, was well tolerated. To our knowledge, this study had recorded the longest median survival time (338 days) reported in cats with mediastinal lymphoma and being FeLV-antigen-positive. In other studies, FeLV-antigen-positive cats had poor prognoses for feline lymphoma, with a median survival time of 37–134 days [10,18,28]. On the other hand, LOPH chemotherapy was well tolerated in a recent study, with a median survival time of 214 days [19].

Of all the variables analyzed, COP response and thoracocentesis after COP significantly affected survival. Cats with a complete response (MST 379 days) had highly significantly longer survival times than those with a partial response (MST 134 days) or no response (MST 121 days), which was similar to other reports [4,7,8,18,19,21]. Cats that required thoracocentesis after COP (MST 147 days) had shorter survival times than those did not require thoracocentesis after COP (MST 373 days). If thoracocentesis had been performed after COP, the cat had a partial or no response to COP. However, COP response and thoracocentesis after COP, as prognostic factors, can only be assessed after treatment.

A prognostic factor parameter at the onset was that cats aged <4 years (MST 373 days) had longer survival times than cats aged at least 4 years old (MST 212 days). Other studies have shown that mediastinal lymphoma develops in young-aged cats [7,18,25] but there is no available information on FeLV-antigen-positive cats. The mechanism of FeLV-antigen-positive cats causing lymphoma is through the insertion of a provirus at many different sites in the host’s genome near a cellular oncogene (most commonly *myc*) [14,29,30,31]. This finding should be investigated further in terms of clinical outcomes in feline lymphoma with FeLV-antigen-positive status.

Through multivariable analysis of hematologically adverse events, cats with anemia before COP, with azotemia after the 2nd induction, and with elevated ALT after the 1st induction, had an increased hazard of death. Surprisingly, blood transfusion during treatment produced no significant change in survival time. FeLV-antigen-positive cats were found in considerable numbers (92 cats) in this study, perhaps due to FeLV infection pathophysiology, which includes bone marrow disorders (mostly anemia), neoplasia (mostly lymphoma) and immunosuppression, leading to susceptibility to secondary infections [14,32]. Elevated ALT after induction could indicate a disorder characterized by the liver’s inability to metabolize substances in the body, as well as azotemia, which is defined as metabolic abnormalities caused by tumor cell cytolysis that occurs spontaneously or as a result of therapy. These results are similar to those from other studies [33,34].

Because of its retrospective character, this study had several limitations. Full clinical signs and diagnostic work-up were not available for all cats. The staging of lymphoma was not identified. Only mediastinal or mediastinal plus other sites lymphoma were included. Therefore, further study may include others form of lymphoma, as well as non-infected FeLV cats.

## 5. Conclusions

This study showed that COP chemotherapy was well tolerated in FeLV-antigen-positive cats and resulted in compatible survival to non-infected cats. Based on weekly monitoring of clinical signs and adverse events until after the 3rd induction of COP chemotherapy, there were no life-threatening events that resulted in death. Prognostic factors were cat age at diagnosis and hematological toxicities. Cats under the age of 4 years had longer survival times than those aged at least 4 years. Anemia (before COP), azotemia (after the 2nd induction) and elevated ALT (after the 1st induction) had an increased hazard of death.

## Figures and Tables

**Table 1:** Patient characteristics.

Variable	*n*	%
Age (yr)		
<1	8	8.70
1–2	35	38.04
2–3	20	21.74
3–4	15	16.30
>4	14	15.22
Sex		
Male	62	67.39
Female	30	32.61
Breed		
Domestic Shorthairs	88	95.65
Others (Scottish fold, Persian)	4	4.35
Reproductive status		
Neutered	56	60.87
Intact	30	32.61
Unknown	6	6.52
Anatomical location		
Mediastinal	78	84.78
Mediastinal + others sites	14	15.22
Thoracocentesis before COP		
Yes	65	70.65
No	27	29.35
Thoracocentesis after COP (times)		
0	69	75.00
1	16	17.39
>1	7	7.61
Blood transfusion before 4th week		
Yes	6	6.52
No	86	93.48
Blood transfusion after 4th week		
Yes	10	10.87
No	82	89.13
COP response		
Complete	75	81.52
Partial	14	15.22
No	3	3.26

COP, cyclophosphamide, vincristine, and prednisolone.

**Table 2:** Clinical signs of feline lymphoma on day of diagnosis, after 1st, 2nd, and 3rd induction of COP chemotherapy.

Clinicopathological Sign		Before COP		After 1st Induction		After 2nd Induction		After 3rd Induction	
		No. Cats	%	No. Cats	%	No. Cats	%	No. Cats	%
Respiratory disorders	Score 1	54/92	58.70	14/92	15.22	0/92	0.00	1/91	1.10
	Score 2	12/92	13.04	0/92	0.00	1/92	1.09	0/91	0.00
Loss of appetite	Score 1	27/92	29.35	19/92	20.65	12/92	13.04	4/91	4.40
	Score 2	8/92	8.70	1/92	1.09	1/92	1.09	0/91	0.00
Lymphadenomegaly	Score 1	5/53	9.43	5/66	7.58	3/65	4.62	0/71	0.00
	Score 2	5/53	9.43	3/66	4.55	3/65	4.62	0/71	0.00
Asthenia postration	Score 1	19/92	20.65	5/92	5.43	3/92	3.26	1/91	1.10
	Score 2	4/92	4.35	0/92	0.00	0/92	0.00	0/91	0.00
Dehydration	Score 1	20/92	21.74	8/92	8.70	7/92	1.00	1/91	1.10
Weight loss	Score 1	9/92	9.78	22/92	23.91	11/92	11.96	7/91	7.69
	Score 2	4/92	4.35	2/92	2.17	1/92	1.09	1/91	1.10
Oral lesions	Score 1	2/43	4.65	0/42	0.00	0/41	0.00	0/43	0.00
	Score 2	3/43	6.98	3/42	7.14	1/41	2.44	0/43	0.00
Neurologic disorders	Score 1	1/92	1.09	0/92	0.00	0/92	0.00	0/91	0.00
	Score 2	2/92	2.17	1/92	1.09	1/92	1.09	1/91	1.10
Conjunctivitis		1/92	1.09	0/92	0.00	1/92	1.09	0/91	0.00
Skin lesions		0/92	0.00	1/92	1.09	0/92	0.00	0/91	0.00
Pale mucous membranes		4/92	4.35	6/92	6.52	5/92	5.43	4/91	4.40
Polyuria/Polydipsia		0/92	0.00	0/92	0.00	0/92	0.00	0/91	0.00

**Table 3:** Adverse events related to COP chemotherapy in cats.

Adverse Events		Before COP		After 1st Induction		After 2nd Induction		After 3rd Induction	
		No. Cats	%	No. Cats	%	No. Cats	%	No. Cats	%
Anemia	Grade I	7/92	7.61	25/92	27.17	23/91	25.27	15/90	16.67
	Grade II	4/92	4.35	9/92	9.78	14/91	15.38	16/90	17.78
	Grade III	1/92	1.09	4/92	4.35	2/91	2.20	1/90	1.11
	Grade IV	0/92	0.00	1/92	1.09	1/91	1.10	0/90	0.00
Leukopenia	Grade I	1/92	1.09	12/92	13.04	18/91	19.78	7/90	7.78
	Grade II	0/92	0.00	2/92	2.17	1/91	1.10	1/90	1.11
Neutropenia	Grade I	0/89	0.00	3/86	3.49	5/89	5.62	5/88	5.68
	Grade II	0/89	0.00	1/86	1.16	2/89	2.25	1/88	1.14
	Grade III	0/89	0.00	0/86	0.00	1/89	1.12	0/88	0.00
sCr	Grade I	28/86	32.56	14/88	15.91	10/84	11.90	14/88	15.91
	Grade II	2/86	2.33	0/88	0.00	0/84	0.00	1/88	1.14
	Grade III	1/86	1.16	0/88	0.00	0/84	0.00	0/88	0.00
ALT	Grade I	1/86	1.16	4/86	4.65	1/81	1.23	3/88	3.41
	Grade II	0/86	0.00	0/86	0.00	1/81	1.23	0/88	0.00
	Grade III	0/86	0.00	0/86	0.00	0/81	0.00	2/88	2.27
	Grade IV	3/86	3.49	4/86	4.65	3/81	3.70	3/88	3.41
Anorexia	Grade I	22/92	23.91	15/92	16.30	9/92	9.78	4/91	4.40
	Grade II	7/92	7.61	6/92	6.52	3/92	3.26	1/91	1.10
	Grade III	6/92	6.52	3/92	3.26	2/92	2.17	1/91	1.10
Vomiting	Grade I	7/92	7.61	5/92	5.43	6/92	6.52	4/91	4.40
	Grade II	6/92	6.52	0/92	0.00	0/92	0.00	1/91	1.10
Diarrhea	Grade I	0/92	0.00	1/92	1.09	2/92	2.17	4/91	4.40

COP, cyclophosphamide, vincristine, and prednisolone; sCr, serum creatinine; ALT, alanine aminotransferase.

**Table 4:** Cox-regression analysis of 76 cats treated with COP chemotherapy.

Factor	Hazard Ratio	95% CI	*p*
Age	2.6	1.2–5.7	0.017 *
Sex	0.81	0.42–1.6	0.530
Status	1.2	0.72–2	0.500
Anatomical location	1	0.45–2.2	0.990
Thoracocentesis before COP	0.75	0.41–1.4	0.350
Thoracocentesis after COP	2.5	1.4–4.7	0.002 *
Blood transfusion before 4th induction	1.5	0.51–4.1	0.480
Blood transfusion after 4th induction	0.88	0.4–2	0.760
COP response	2.1	1.2–3.7	0.009 *
Anemia before COP	2.5	1.2–5.3	0.013 *
Anemia after 1st induction	1.5	0.84–2.5	0.180
Anemia after 2nd induction	1.3	0.76–2.3	0.330
Anemia after 3rd induction	0.88	0.49–1.6	0.680
Leukopenia before COP	3.4	0.46–26	0.230
Leukopenia after 1st induction	1.1	0.51–2.2	0.880
Leukopenia after 2nd induction	1.8	0.94–3.4	0.075
Leukopenia after 3rd induction	0.63	0.23–1.8	0.390
Neutropenia before COP	NA	NA	NA
Neutropenia after 1st induction	1.3	0.39–4.1	0.700
Neutropenia after 2nd induction	0.92	0.36–2.3	0.870
Neutropenia after 3rd induction	0.36	0.087–1.5	0.160
SCr before COP	0.65	0.35–1.2	0.180
SCr after 1st induction	1.9	0.96–3.9	0.065
SCr after 2nd induction	2.3	1–5.2	0.044 *
SCr after 3rd induction	1.3	0.66–2.5	0.450
ALT before COP	2.3	0.84–6.6	0.110
ALT after 1st induction	2.8	1.2–6.2	0.014 *
ALT after 2nd induction	1.1	0.33–3.5	0.910
ALT after 3rd induction	1	0.37–2.8	0.970

COP, cyclophosphamide, vincristine, and prednisolone; sCr, serum creatinine; ALT, alanine aminotransferase; NA, not available; *, statistical differences (*p* < 0.05).

## Data Availability

The data presented in this study are available within the article. Raw data supporting this study are available from the corresponding author.

## Supplementary Materials

The following supporting information can be downloaded at: https://www.mdpi.com/article/10.3390/ani12070900/s1, Table S1: Clinical signs of feline lymphoma on day of diagnosis, after 1st, 2nd and 3rd induction of COP chemotherapy; Table S2: Adverse events related to COP chemotherapy in cats.
