# Supplementary material for: Effectiveness and Adverse Events of Cyclophosphamide, Vincristine, and Prednisolone Chemotherapy in Feline Mediastinal Lymphoma Naturally Infected with Feline Leukemia Virus
Source: Animals (Basel). 2022 Mar 31;12(7):900. doi: 10.3390/ani12070900 (PMC8997098; doi:10.3390/ani12070900)
Supplement: Supplementary file 1 [file animals-12-00900-s001.zip › animals-1631019-supplementary.pdf]

Table S1. Clinical signs of feline lymphoma on day of diagnosis, after 1st, 2nd and 3rd induction of COP chemotherapy.

**Clinicopathological sign**	**Score**		
	**0**	**1**	**2**
Respiratory disorders	No	Mild	Severe
Loss of appetite	No	Partial	Total
Lymphadenomegaly	No	Localized	Generalized
Asthenia postration	No	Slightly	Severe
Dehydration	No	< 10%	> 10%
Weight loss	No	Thinness	Cachexia
Oral lesions	No	Mild	Severe
Neurologic disorders	No	Ataxia	Seizure
Conjunctivitis	No	Yes	
Skin lesion	No	Yes	
Pale mucous membrane	No	Yes	
Polyuria/Polydipsia	No	Yes	

COP, cyclophosphamide, vincristine, and prednisolone.

Table S2. Adverse events related to COP chemotherapy in cats.

**Adverse event**	**Grade**				
	**I**	**II**	**III**	**IV**	**V**
Anemia (%)	25-29	20-24.9	15-19.9	<15	Death
Leukopenia (x10^3/cumm)	3.0-5.0	1-2.99	0.5-0.99	<0.5	Death
Neutropenia (x10^3/cumm)	1,500-2,500	1,000-1,499	500-999	<500	Death
sCr (mg%)	1.61-2.2	2.21-2.8	2.81-5.0	>5.0	Death
ALT (37°C IU/L)	140-280	281-350	351-420	>420	Death
Vomit	<3 episode in 24 h	3-10 episodes in 24 h; parenteral fluids (IV or SC) indicated ≤48 h; medications indicated	Multiple episodes >48 h and IV fluids or PPN/TPN indicated >48 h	Life-threatening	Death
Diarrhea	Fecal score 4/7	Fecal score 5-6/7; parenteral (IV or SC) fluids indicated ≤48 h	Fecal score 7/7; IV fluids >48 h; hospitalization	Life-threatening	Death
Anorexia	Dietary change required to Maintain appetite	Oral intake altered (≤3 days) without significant weight loss	Significant weight loss (≥10%); IV fluids, tube feeding or force feeding indicated	Life-threatening	Death

COP, cyclophosphamide, vincristine, and prednisolone; sCr, serum creatinine; ALT, alanine aminotransferase; IV, intravenous; SC, subcutaneous; PPN, partial parenteral nutrition; TPN total parenteral nutrition.
